# AI-assisted Diagnosis of Nonmelanoma Skin Cancer in Resource-Limited Settings

**DOI:** 10.1158/1055-9965.EPI-25-0132

**Published:** 2025-04-25

**Authors:** Spencer Ellis, Steven Song, Derek Reiman, Xuan Hui, Renyu Zhang, Mohammad Hasan Shahriar, Maria Argos, Mohammed Kamal, Christopher R. Shea, Robert L. Grossman, Aly A. Khan, Habibul Ahsan

**Affiliations:** 1 Departments of Pathology and Family Medicine, University of Chicago, Chicago, Illinois.; 2 Department of Computer Science, University of Chicago, Chicago, Illinois.; 3 Toyota Technical Institute at Chicago, Chicago, Illinois.; 4 Department of Public Health Sciences, University of Chicago, Chicago, Illinois.; 5 Institute for Population and Precision Health, University of Chicago, Chicago, Illinois.; 6 Division of Epidemiology and Biostatistics, University of Illinois at Chicago, Chicago, Illinois.; 7 Department of Pathology, Bangabandhu Sheikh Mujib Medical University, Dhaka, Bangladesh.; 8 Section of Dermatology, Department of Medicine, University of Chicago, Chicago, Illinois.; 9 Section of Biomedical Data Science, Department of Medicine, University of Chicago, Chicago, Illinois.; 10 Center for Translational Data Science, University of Chicago, Chicago, Illinois.; 11 Chan Zuckerberg Biohub Chicago, Chicago, Illinois.

## Abstract

**Background:**

Early and precise diagnosis is vital to improving patient outcomes and reducing morbidity. In resource-limited settings, cancer diagnosis is often challenging due to shortages of expert pathologists. We assess the effectiveness of general-purpose pathology foundation models (FM) for the diagnosis and annotation of nonmelanoma skin cancer (NMSC) in resource-limited settings.

**Methods:**

We evaluated three pathology FMs (UNI, PRISM, and Prov-GigaPath) using deidentified NMSC histology images from the Bangladesh Vitamin E and Selenium Trial to predict cancer subtype based on zero-shot whole-slide embeddings. In addition, we evaluated tile aggregation methods and machine learning models for prediction. Lastly, we employed few-shot learning of PRISM tile embeddings to perform whole-slide annotation.

**Results:**

We found that the best model used PRISM’s aggregated tile embeddings to train a multilayer perceptron model to predict NMSC subtype [mean area under the receiver operating characteristic curve (AUROC) = 0.925, *P* < 0.001]. Within the other FMs, we found that using attention-based multi-instance learning to aggregate tile embeddings to train a multilayer perceptron model was optimal (UNI: mean AUROC = 0.913, *P* < 0.001; Prov-GigaPath: mean AUROC = 0.908, *P* < 0.001). We finally exemplify the utility of few-shot annotation in computation- and expertise-limited settings.

**Conclusions:**

Our study highlights the important role FMs may play in confronting public health challenges and exhibits a real-world potential for machine learning–aided cancer diagnosis.

**Impact:**

Pathology FMs offer a promising pathway to improve early and precise NMSC diagnosis, especially in resource-limited environments. These tools could also facilitate patient stratification and recruitment for prospective clinical trials aimed at improving NMSC management.

## Introduction

Computational pathology (CPath) is rapidly advancing, driven by progress in machine learning image analysis and the increasing availability of high-resolution whole-slide images (WSI). The potential of CPath lies in assisting pathologists through the automation of tasks, including cancer detection and region-of-interest (ROI) identification, tumor subtyping and grading, prognosis prediction, and the discovery of visual and subvisual biomarkers (1–3). CPath methods developed in the past several years have been shown to be highly effective in tasks as varied as grading prostate cancer (4), lymph node metastasis detection (5), and predicting colorectal cancer outcomes (6). However, a major challenge associated with the development of these methods has been the amount of labeled data required to train the models, which requires expert annotation of a large number of WSIs. This becomes infeasible in the case of rare diseases. Additionally, as gigapixel-scale WSIs must be partitioned into patches to be used for model input, there are several limitations associated with their use in machine learning algorithms. First, supervised learning can be extremely challenging as the vast majority of patches will not contain diagnostically relevant data, resulting in poor data efficiency. Second, slide-level predictions require the aggregation of patch-level predictions, often requiring manually developed heuristics (7, 8).

To overcome these challenges, the recent focus within CPath research has been on the development of foundation models (FM) that can generalize across tasks, tissue types, and diseases (arXiv 2405.10254; refs. 9–11). Such FMs are analogous to breakthroughs in natural language processing, in which task-agnostic models have been developed using self-supervised training techniques to output feature representations of sequences of text (12). As self-supervised training does not require data annotations/labels, extremely large quantities of input data can be used, and models can learn from vast, heterogeneous datasets. Efficient self-supervision techniques for image data, such as DINOv2 (12), have, therefore, enabled the development of task-agnostic FMs for CPath, trained on thousands to millions of WSIs (arXiv 2405.10254; refs. 9–11).

These FMs hold particular promise in settings in which access to expert pathologists is constrained, such as in Bangladesh (13, 14). Arsenic contamination of groundwater in Bangladesh is considered to be the largest mass poisoning of a population in history by the World Health Organization, with an estimated 35 to 77 million Bangladeshi people having been chronically exposed to arsenic through drinking water (15–18). Arsenic toxicity is closely dependent on the amount of ingestion, and once consumed, 40% to 60% of the arsenic is retained in the human body and passes slowly into the skin, resulting in malignancies, namely nonmelanoma skin cancer (NMSC; refs. 15, 19). More than 95% of NMSC cases consist of basal-cell carcinoma (BCC) and cutaneous squamous cell carcinoma (SCC). The former is a slow-growing, locally invasive epidermal tumor. The latter arises from dysplastic epidermal keratinocytes. SCC can be either *in situ* (Bowen disease) or invasive. Bowen disease is generally considered a low-grade form of SCC, with a reported risk of progression to invasive SCC of up to 3% (20–22). Current evidence supports that the delay in detection is the main underlying cause of aggressive tumor behavior and subsequent morbidity in patients with NMSC (20, 23). Hence, early and precise detection is critical for controlling disease progression and could lead to a substantially higher success rate in treatment. Usually, both types are readily identified by a pathologist in a timely manner, and in this situation, patients would benefit from timely treatment. However, in resource-limited settings, such accurate and timely detection becomes a challenge due to the limited number of expert pathologists, resulting in affected individuals having a poor prognosis. In Bangladesh (and many other countries), as predisposing exposure and susceptibility are difficult to eradicate, people who are chronically exposed to arsenic through consuming contaminated water are deemed at high risk of NMSC and other health consequences (23).

In this study, we evaluated the performance of three FMs: Prov-GigaPath (9), UNI (10), and PRISM (arXiv 2405.10254), for the diagnosis of NMSC, comparing their diagnostic performance against a standard ResNet18 tile encoder baseline (24). Using deidentified NMSC hematoxylin and eosin (H&E)–stained WSIs from the Bangladesh Vitamin E and Selenium Trial (BEST), collected by the Institute for Population and Precision Health at the University of Chicago, we evaluated each FM’s ability to classify NMSC subtypes at the whole-slide level. This involved extracting tile embeddings from the FMs, aggregating them using standard methods such as global average pooling (GAP) and attention-based deep multiple instance learning (ABMIL; ref. 25), as well as FM-specific aggregation techniques. These aggregated slide-level embeddings were then input into downstream classifiers [logistic regression (LR), XGBoost (26), or a shallow multilayer perceptron (MLP)] for final subtype prediction. Details of these methods are provided in Table 1.

**Table 1.** Important concepts and terms used in this study.

**FMs**	
Prov-GigaPath	Prov-GigaPath is a vision model developed by Microsoft, consisting of a tile encoder and a slide aggregator, and was trained on 171,189 WSIs. Its “tile encoder” is a vision transformer (ViT) with 1.1 billion parameters, trained using the DINOv2 self-supervised learning algorithm. Its “slide aggregator” is a LongNet (a transformer that uses dilated attention for long sequences) trained using masked autoencoding. Slide-level embeddings are produced either by GAP over the contextualized tile embeddings output by the LongNet or by using the embedding of a specialized [CLS] token.
UNI	UNI is a vision model developed by the Mahmood Lab at Brigham and Women’s Hospital, consisting only of a tile encoder, and was trained on more than 100,000 WSIs. Its “tile encoder” is a ViT with 303 million parameters, trained using DINOv2.
PRISM	PRISM is a vision-language model developed by Paige, consisting of a tile encoder and a slide aggregator. For our study, we only use the vision components of the model. Its “tile encoder” is the Virchow model, also developed by Paige, a ViT with 631 million parameters trained using DINOv2 on 1.5 million WSIs. Its “slide aggregator” is a perceiver model trained jointly with a text model on more than 587 WSIs using both contrastive and generative objectives. Slide-level embeddings are produced by using the embedding of a specialized [CLS] token.
**Aggregation methods**	
GAP	GAP produces a single slide-level embedding by averaging all tile embeddings.
ABMIL	ABMIL aggregates tile embeddings by creating a weighted average embedding. Weights are determined using a learned attention mechanism.
Model-specific slide aggregator	Both Prov-GigaPath and PRISM have model-specific slide aggregators, described above.
**Classification methods**	
LR	Multinomial LR, or softmax regression, applies LR to classification problems with more than two classes.
XGBoost	XGBoost is a gradient boosting algorithm that uses an ensemble of multiple simple decision trees to generate predictions.
MLP	An MLP is a feedforward, fully connected neural network. In our case, we use a network with three hidden layers of dimensions 1,024, 512, and 256 and a final classification layer of dimension 4.

## Materials and Methods

### Data source

This study utilized 2,130 H&E WSIs of 553 NMSC biopsies from 455 patients. These samples were collected by the BEST. BEST is a 2 × 2 factorial randomized prevention trial of 6-year daily supplementation of vitamin E and selenium among 7,000 participants from two regions (Araihazar and Matlab) in Bangladesh (27). NMSC was ascertained at each biennial in-person follow-up exam of all BEST participants who had undergone three levels of evaluation (first by research physicians, then by senior physicians, and finally by an expert dermatologist) before biopsies were conducted on their suspicious lesions. Smaller lesions (diameter <5 mm) were punch-biopsied, and larger lesions (diameter ≥5 mm) were excised. Among individuals with multiple biopsy-eligible lesions, the lesion most concerning for malignancy was biopsied. When an individual developed lesions at different time points, biopsies were collected at each time point. Formalin-fixed biopsy tissues were processed at a single specialized surgical pathology laboratory in Dhaka, Bangladesh, and processed into H&E slides. Each biopsy generated an average of four slides. We note that the overall quality of these slides was generally below that required in accredited diagnostic pathology labs in the United States. These slides were transported to the University of Chicago Human Tissue Resource Center Core Facility (RRID: SCR_019199), scanned by a Leica Aperio ScanScope XT at 20× magnification (0.5 MPP), and saved in the SVS format for analysis. The final dataset we used in our experiments contains a majority of benign cases with Bowen disease as the next most prevalent class, followed by BCC, and finally invasive SCC (Table 2). All data were collected with approval from the Institutional Review Boards of the University of Chicago, locally from the Bangladesh Medical Research Council, and from the International Centre for Diarrhoeal Disease Research, Bangladesh, in accordance with the guidelines set by the US Common Rule and the Declaration of Helsinki. All participants provided written informed consent. Patients with identified tumors were provided standard of care through the UChicago Research Bangladesh Community Hospital, as well as through regional tertiary hospitals with referrals.

**Table 2.** Dataset characteristics broken down by NMSC subtype at the individual patient level and the biopsy level.

Data characteristics					
NMSC Type	Benign	Bowen	BCC	SCC	Total
Patients, *n* (%)	210 (46)	140 (31)	85 (19)	20 (4)	455 (100)
Biopsies, *n* (%)	229 (41)	171 (31)	116 (21)	37 (7)	553 (100)

NOTE: Patients with multiple biopsies were assigned the most frequent cancer type across all their biopsies.

### Ascertainment of NMSC

All WSIs were given a diagnosis by a dermatopathologist (C.R. Shea) from the Section of Dermatology at the University of Chicago Medical Center. The diagnosis included whether there was an indication for NMSC and, if so, the histopathologic subtype of NMSC. Of note, our specimen bank did not include any actinic keratoses; this may reflect the generally dark skin pigmentation (Fitzpatrick types V and VI) of the Bangladeshi study population, in which actinic keratoses are rare.

### Data preprocessing

For input into the FMs, the WSIs were preprocessed into collections of nonoverlapping 256 × 256 tiles. We utilized Prov-GigaPath’s preprocessing toolbox, which segmented each WSI into foreground and background using Otsu’s method (28) to determine a threshold for foreground pixels based on average luminance for each pixel across the channel dimension and a fixed tile occupancy threshold of 0.1. Tiles with occupancy below the threshold were discarded to ensure that only tiles containing tissue were retained and used in downstream tasks. Preprocessing of the 2,130 WSIs resulted in 4,316,353 image tiles.

### Framework

**Figure 1.**
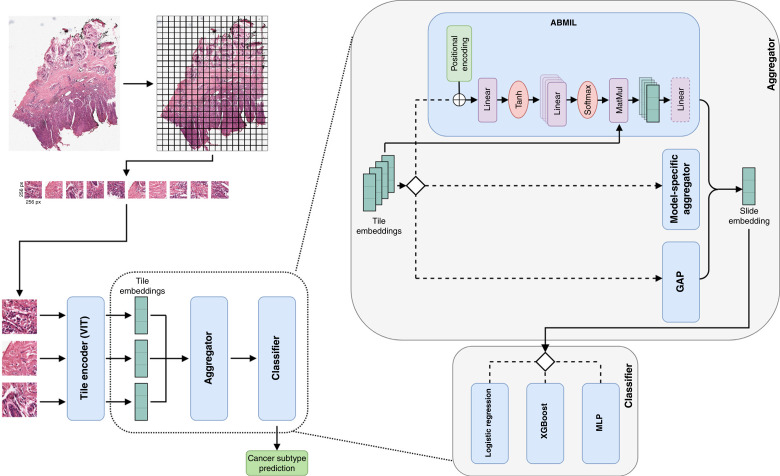
Overview of generating whole-slide embeddings using FMs. Each WSI is split into 256 × 256 px tiles, and tiles with visible tissue are selected for further processing. Tile features are extracted by a tile encoder, tile embeddings for a WSI are aggregated into a slide embedding, and NMSC is classified using the slide-level embedding. Tile aggregation methods include ABMIL, FM-specific aggregators, or GAP. Classification is performed using LR, XGBoost, or a shallow MLP. ViT, vision transformer.

To obtain a slide-level classification, FM tile encoders were first used to generate tile embeddings. Each FM’s tile encoder was run according to the specific model’s documentation, including prescribed image transformations. For comparison with ResNet18 as a tile encoder, tiles were center-cropped to 224 px squares per channel, normalized using ImageNet values, and embedded using ResNet18 pretrained on ImageNet. For FMs that included an additional slide aggregator (Prov-GigaPath and PRISM), slide embeddings were generated using the methods outlined in their respective documentation. For Prov-GigaPath, two sets of slide embeddings were generated: one using the [CLS] token from the final model layer and the other using GAP (which we refer to as “pool”) of all contextualized tile embeddings output by the final model layer. For each FM tile encoder, we additionally develop our own slide aggregators over precomputed tile embeddings. The simplest of these is GAP of tile embeddings into a single-slide embedding. Alternatively, we train a joint aggregator–classifier using ABMIL and a three-layer MLP classifier. Lastly, for each slide aggregator, the slide embeddings are used to train a multinomial LR, XGBoost, and a three-layer MLP classifier. Our overall classification framework is outlined in Fig. 1. For all FM tile encoders and slide aggregators, we use the models as is with no additional fine-tuning, only training models over the frozen tile- or slide-level embeddings.

As the output of our pipeline resulted in slide-level classifications, although pathologist labels were given at the biopsy level, we produced biopsy-level classifications by averaging predicted slide-level cancer-type probabilities across all slides for each biopsy.

All deep learning models we studied were implemented using Python 3.10.14 (RRID: SCR_008394) and PyTorch 2.4.1 (RRID: SCR_018536). For ABMIL- or MLP-based models, a maximum of 30 epochs were used for training with gradient accumulation over 16 slides. A learning rate of 1e−5 was used with an AdamW optimizer over cross-entropy loss of pathologist-labeled diagnoses. Graphics processing unit (GPU)-accelerated inference of each FM tile encoder could be done using a single NVIDIA 2080 Ti, a consumer-grade GPU from 2018.

### Aggregation methods

Typically, feature extraction methods that output whole-image classifications rely on consistent input sizes in terms of image sizes and the number of image tiles. For WSIs, their size, shape, and number of foreground tiles can vary drastically; in the BEST dataset, foreground tile counts from a single WSI ranged from 82 to 38,404 tiles. This presents a major challenge to the efficient classification of WSIs. To overcome this, we considered three aggregation methods: GAP, ABMIL, and FM-specific aggregators.

GAP is the simplest of these methods. Tile embeddings for a given WSI were stacked as row vectors into a single matrix. The slide embedding is then given by uniformly averaging the tile embeddings across all rows. An implicit assumption of GAP is that all tile embeddings have equal importance.

ABMIL challenges this by computing a weighted average of tile embeddings, assigning “importance” to each tile depending on its usefulness for the model in determining a diagnosis. ABMIL learns this weighting according to the specific data it is trained on, unlike naïve aggregation methods such as GAP. Additionally, visualization of the tile weighting, via an attention heatmap, helps provide model explainability. One specific requirement of ABMIL is representing the 2D layout of tiles in a 1D sequence. In our implementation, we encoded tile positions by adding 2D sinusoidal positional embeddings to the tile embedding vectors prior to input into the MIL module. This is similar to the positional embedding used by Prov-GigaPath. Experiments using ABMIL included both gated and ungated implementations (in which gated attempts to boost model expressiveness), as well as 1 or 8 parallel attention branches, in which 8 was chosen to match the original transformer implementation with 8 attention heads (25, 29). A fully connected layer was then used to project the attention output to the original input embedding dimension. We trained each ABMIL module jointly with an MLP classification head to provide a well-defined training objective.

Both Prov-GigaPath and PRISM include slide aggregators for use in downstream slide-level tasks, which we used directly as prescribed in each model’s documentation. Prov-GigaPath uses a LongNet (arXiv 2307.02486) vision transformer architecture for its slide aggregator, utilizing a resource-efficient dilated self-attention mechanism to control for long tile sequence lengths. The LongNet produces contextualized tile embeddings, gathering relevant information from each input tile embedding and the rest of the tiles in the sequence. The final slide-level embedding is either the [CLS] token or the average output contextualized tile embedding from the output of the final layer. PRISM uses a perceiver architecture for its slide aggregator. The perceiver architecture handles variable sequence length by projecting input into a fixed-length latent sequence through a cross-attention module that uses a latent query matrix parameterized by the model (30). PRISM utilizes a latent dimension of 512, plus an additional dimension for the [CLS] token. The final slide-level embedding is the [CLS] token output from the final layer of the perceiver.

### Classification methods

To focus on resource-constrained environments, we explored popular, resource-efficient classification methods for the task of NMSC classification: multinomial LR, XGBoost, and a shallow MLP. For LR, we used LogisticRegression from scikit-learn (RRID: SCR_002577) with the SAGA solver, L2 regularization, and 1,000 maximum iterations. For XGBoost, we used XGBClassifier from XGBoost (RRID: SCR_021361) with default hyperparameters and a softmax objective. Finally, the shallow MLP was implemented with three hidden layers of dimensions 1,024,512, and 256, regardless of the input dimension to the MLP. MLP models were implemented as described above. The number of layers and their dimensions were chosen based on empirical experiments to balance model complexity and performance.

### Fivefold cross-validation and training

All experiments were repeated using fivefold cross-validation, with four folds used for training and one fold used for validation. As multiple biopsies were taken from some patients, it was vital to ensure that there was no patient-specific data leakage between folds. Folds were, therefore, constructed by splitting the full dataset by patient and stratifying by the target variable (i.e., NMSC classification). This ensures that all data from a single patient are contained within a single fold, preventing data leakage between training and validation sets. The same folds were used for all experiments.

For experiments in which slide-level embeddings were available, we trained the MLP, LR, and XGBoost classifiers using the embeddings as is, with no transformations or normalization applied. For experiments in which we also trained an aggregator, the aggregator was trained end to end with the MLP classification head, and all training was performed using unmodified tile embeddings with no transformations or normalization applied.

### Evaluation

Evaluation was conducted using the area under the receiver operating characteristic curve (AUROC). Metrics were calculated using a one-vs-rest strategy for each cross-validation fold on the fold reserved for validation. This resulted in classification metrics for each cancer type on each fold, for a set of 20 results per experiment. Pairwise comparison of experiment results was performed using a one-sided Wilcoxon signed-rank test. A weighted mean AUROC was calculated by taking the sum of the individual AUROC values from one-vs-rest evaluations, weighted by the respective class proportion.

### Attention heatmap visualization

For slide aggregators that utilize an attention mechanism, the attention scores from the final layer were extracted from the models to produce heatmap visualizations of the attention scores. These heatmaps were overlaid on the original WSIs. This highlights regions of the WSI that each model considers most important for its classification decision, providing interpretability to a model’s predictions.

### Few-shot framework

For our few-shot annotation framework, we first derived representative tiles for the types of tissue we aim to classify. Specifically, we derived representative tiles for invasive SCC, Bowen disease, superficial BCC, nodular BCC, epidermis, dermis, subcutis, and artifact. These classes are an extension of the four classes used in the zero-shot framework due to the stark difference in appearance of tissue between, for example, dermis and epidermis. As a result, the use of seven classes enables the generation of finer-grained representative embeddings. Representative tiles for these seven classes were extracted from manually annotated ROIs across five samples spanning the different cancer types. The ROIs were broadly annotated as quadrangular bounding boxes surrounding the tissues of interest. The ROIs were annotated by a medical student and reviewed by an expert dermatopathologist (C.R. Shea).

We extract tiles from each ROI and embed each tile using the tile encoder for a given FM. As the number of representative tiles per ROI depends on the size of the ROI, we apply GAP to all extracted tile embeddings across each class to derive one representative embedding per class. To do few-shot annotation at inference time, we classify tiles from the sample WSI as the class in which the representative embedding is most similar to the inference tile embedding, as measured by the dot product between embeddings.

### Data availability

The code developed for this study is publicly available on GitHub: https://github.com/sjne09/akdslab-skin-cancer. Due to patient privacy concerns related to a vulnerable population, the WSI dataset cannot be made publicly available. However, data access may be granted upon reasonable request to the corresponding author and is subject to appropriate data use agreements.

## Results

**Figure 2.**
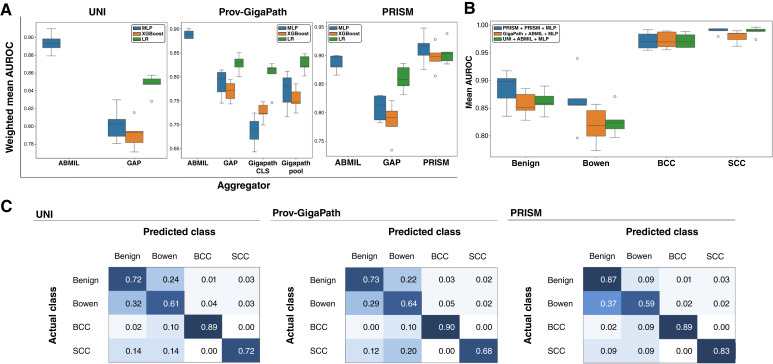
Evaluation of FM performance for classifying skin cancer types. **A,** Classification results with different combinations of tile aggregation and classifier models for each FM. **B,** Per-class classification results using the best combination of each FM. **C,** Confusion matrices for each model.

We first sought to identify the optimal combination of tile embedding aggregation strategy and downstream classification model for each pathology FM evaluated (Fig. 2A). We observe that for UNI- and Prov-GigaPath-derived tile embeddings, ABMIL aggregation with MLP classification attains the highest weighted mean AUROC across all test splits (UNI: mean AUROC = 0.913, *P* < 0.001; Prov-GigaPath: mean AUROC = 0.908, *P* < 0.001). For PRISM, an MLP trained over PRISM’s own tile aggregator outperforms all other methods tested for PRISM tile embeddings (mean AUROC = 0.925, *P* < 0.001). Additionally, the best-performing combinations for each FM significantly outperformed a baseline model of ResNet18 trained on the BEST dataset (mean AUROC = 0.805, *P* < 0.001). Notably, across all FM’s GAP aggregated embeddings or over Prov-GigaPath’s provided tile aggregator, the computationally simplest classification model using LR does better than MLP or XGBoost (UNI + GAP + LR: mean AUROC = 0.847; Prov-GigaPath + GAP + LR: mean AUROC = 0.828; Prov-GigaPath + GigaPathPool + LR: mean AUROC = 0.827; Prov-GigaPath + CLS + LR: mean AUROC = 0.803; PRISM + GAP + LR: mean AUROC = 0.860). Although the combinations with LR lag behind ABMIL/MLP, there is a surprisingly small performance gap.

Next, we compared the subtype-specific classification performance using the optimal aggregator/classifier combination identified for each FM (Fig. 2B). We observe that for benign classification, PRISM performs the best, whereas for BCC classification, all models perform comparably. However, for SCC classification, Prov-GigaPath lags behind PRISM and UNI. Yet UNI is worse than PRISM at distinguishing Bowen disease.

To further investigate these performance differences, we generated confusion matrices for each FM’s optimal configuration (Fig. 2C). We observe that PRISM has the least confusion on actual benign samples. Additionally, the same is true for PRISM on actual SCC samples. Especially given the relationship between Bowen disease and SCC, we observe that PRISM predicts the fewest Bowen disease cases for invasive SCC samples. This may help explain PRISM’s strong performance in distinguishing between Bowen and SCC cases compared with the other FMs. However, all FMs demonstrated some degree of confusion between Bowen disease and benign cases.

**Figure 3.**
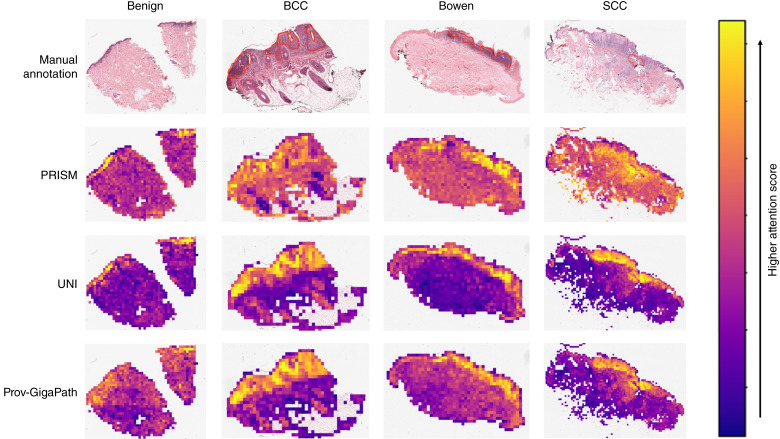
Attention heatmap annotations for the best-performing tile aggregator of each FM, compared against expert pathologist-annotated ROIs in red. Annotations are shown for benign, BCC, Bowen, and SCC slides.

To explore the mechanisms underlying PRISM’s strong performance, we visualized tile-level importance using attention maps generated by each model’s respective optimal tile aggregator (Fig. 3). We extracted the attention maps of each model for the slides of benign tissue, BCC, Bowen disease, and SCC. We observed that the attention of the Prov-GigaPath and UNI aggregators focuses more on the epithelium while paying relatively less attention to the dermis. PRISM’s aggregator still focuses on epidermal regions, especially those that contain cancerous tissue; however, it also pays greater attention to the whole-tissue sample. From an expert dermatopathologist’s perspective, such a broad view is necessary for distinguishing SCC from Bowen disease as dermal invasion can be an important distinguishing feature between the two. Qualitatively, we observed the highest concordance between expert annotations and model attention for PRISM.

**Figure 4.**
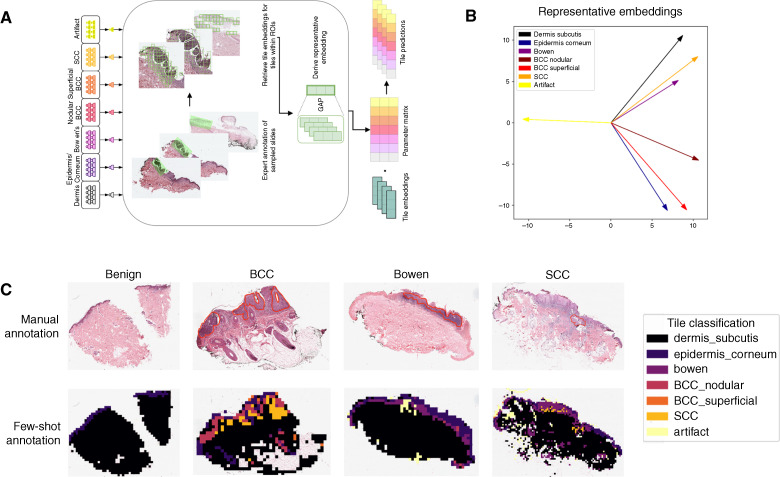
Few-shot annotation of ROIs using PRISM-derived embeddings. **A,** Overview of the few-shot methodology. For each of the tissue subtypes, a representative specimen/biopsy is selected. For each of the selected biopsies, ROIs exhibiting the class of interest are manually annotated; tiles and their embeddings are extracted from these ROIs and averaged to derive a representative embedding per class. At inference time, the dot product between tile embeddings and representative embeddings is used as a similarity score for each class, in which the tile prediction is the class with the greatest similarity. **B,** Visualization of the first two principal components of representative embeddings for each class derived using the few-shot methodology. **C,** Annotated benign, BCC, Bowen, and SCC slides, annotated by an expert pathologist (in red) and by our few-shot framework.

Finally, we evaluated our proposed few-shot annotation framework (Fig. 4). As PRISM tile embeddings performed most optimally, we used PRISM-derived embeddings for our few-shot annotations. After extracting the representative tile embeddings for our seven tissue subclasses, we visualized these representative embeddings by plotting their first two principal components (Fig. 4B). We observed a wide separation of artifact embeddings and a stratification of tissue embeddings that seem to follow the dermatologic organization of the tissue types. For example, invasive SCC’s embedding is sandwiched between the Bowen and dermis/subcutis embeddings, reflecting its relationship with those tissue types.

Using these representative embeddings, we generated few-shot annotations for the same representative slides previously analyzed (benign, BCC, Bowen, SCC; Fig. 4C). We find that our few-shot annotations correctly identify tiles of each cancer type in their respective slides, aligning with expert annotations of these same ROIs. However, we also observe misidentification of tiles. In SCC, many tiles are classified as Bowen; although this may be expected to confound these two classes, the identification of SCC in the BCC slide is less explainable. Despite these inaccuracies, the framework effectively highlighted relevant ROIs containing the misclassified tiles, demonstrating its potential for rapidly identifying areas requiring closer inspection.

## Discussion

Foundation models have revolutionized the field of computational biology, notably in the domains of protein structure prediction, modeling of the genomic landscape, and precision medicine (30–34). This study demonstrates the successful application of CPath FMs for NMSC classification and annotation using whole-slide histology images, establishing their superiority over a traditional convolutional neural network baseline (ResNet18). Leveraging NMSC data from the BEST cohort, we evaluated the zero-shot classification performance of state-of-the-art pathology FMs.

Our results indicate that although all evaluated FMs performed well, architectural nuances and training strategies influence performance on specific tasks. When using ABMIL aggregation on tile embeddings from UNI and Prov-GigaPath (both using visual transformers pretrained with DINOv2), performance was comparable, suggesting that within this specific evaluation framework, differences in training data scale or model parameters between these two FMs did not translate to significant performance gains for NMSC classification. However, PRISM, utilizing its distinct perceiver-based architecture and native aggregation mechanism, achieved significantly higher overall performance. Analysis of attention heatmaps provided a potential explanation: PRISM’s tendency to integrate information from broader tissue regions, including the dermis, likely facilitated better discrimination between NMSC subtypes, particularly the challenging distinction between Bowen disease and invasive SCC, in which stromal context is informative. We hypothesize that this broader attention pattern may stem from PRISM’s multimodal training objective (incorporating text alongside images), which potentially encourages the model to learn more globally relevant features useful for tasks beyond simple classification, such as report generation (35, 36). This observation underscores the potential benefits of developing FM architectures that effectively integrate multimodal data to model complex biological systems. An additional benefit observed was the FMs’ ability, particularly demonstrated in the few-shot setting, to implicitly handle data quality issues by distinguishing tiles containing histologic artifacts.

Despite the overall strong performance, the analysis of misclassification patterns revealed nuances. SCC, the least prevalent subtype in our dataset (4%), exhibited very few false positive predictions, with minimal false negatives (misclassified as benign or Bowen). Conversely, considerable confusion was observed between Bowen disease and benign cases. Pathologically, diagnosing Bowen disease requires identifying full-thickness epidermal keratinocytic atypia, which can be focal. Therefore, sampling variability in biopsies or inherent diagnostic subtlety in borderline cases (e.g., distinguishing Bowen from arsenical keratosis with atypia, classified as benign here) may contribute to this specific confusion. Addressing such nuanced classification challenges might require finer-grained, tile-level supervision or models designed for enhanced interpretability, rather than relying solely on slide-level labels.

We also stress how our few-shot annotation framework can be useful in computation- and expertise-limited settings. First, the ROIs that drive the few-shot representative examples were derived from clear cases of each cancer type, allowing a medical student with limited training to perform the initial manual annotation. Second, as the tile annotation uses a simple vector dot product between embeddings, the few-shot annotation does not require any additional training of a classifier model; no additional data are required, and even simple models such as LR are not needed. The only additional computation required would be for extracting tile embeddings using pretrained FMs, a task that is suitable for relatively inexpensive, consumer-grade hardware.

From a user perspective, we propose using few-shot annotations to aid personnel who must initially sort or process samples, akin to a technician or regional doctor without extensive pathology training. When presented with the few-shot annotation and original H&E, the annotation could assist personnel in identifying ROIs and making a preliminary classification. In situations where the human decision and the annotations differ, such cases can be escalated to a centralized regional facility where an expert pathologist can make a final classification. This would reduce the burden on the experts, who may be limited in number and time, relying on them only for confusing or difficult cases.

Indeed, an automated system for detecting and visualizing histopathologic patterns of NMSC has a wide variety of applications in clinical settings. Considering the quick inference times of both zero- and few-shot applications, these FMs could be integrated into existing clinical management systems to automatically annotate histopathologic patterns on slides and provide complementary diagnostic opinions on challenging cases. This could expedite the diagnostic process for the pathologist, freeing up more time for them to focus on a larger number of cases and making cancer detection more accessible in areas where medical resources are in shortage.

Although our study demonstrates promising results, there are several limitations to consider. First, our primary dataset is derived from a single cohort (BEST), which may limit the generalizability of our findings to other populations. Second, although we discuss the potential clinical utility of our approach, we acknowledge that further research is needed to fully evaluate its integration into the existing clinical workflow in resource-limited settings. This includes addressing practical considerations such as the availability of digital pathology infrastructure. Although the availability of slide scanners in remote areas of low- or middle-income countries is a key barrier to implementing diagnostic algorithms, such infrastructure is at least available in major cities, including Dhaka, the capital city close to our study population. Patients from remote areas, whose slides could be transported and scanned in regional medical institutions, may still benefit from a diagnostic algorithm, especially for patients whose cancer management could be organized locally.

All data used in this study were deidentified to protect patient privacy. However, it is important to acknowledge the potential risks associated with the use of artificial intelligence models in healthcare, particularly in vulnerable populations. These risks include the potential for algorithmic bias, the need for transparency and explainability in model decision-making, and the importance of ensuring equitable access to the technology. We believe that our study takes a step toward addressing these concerns by demonstrating the potential of FMs to improve cancer diagnosis in resource-limited settings, but further research is needed to fully address the ethical implications of this technology.

In conclusion, our work highlights the important role FMs may play in confronting public health challenges and exhibits a real-world potential for machine learning–aided cancer diagnosis. The advent of large-scale, pretrained FMs, such as PRISM, now provides considerable potential for prospective clinical trials to improve treatment outcomes and benefit patients through early and precise diagnosis, especially in resource-limited settings. We demonstrate that FMs can outperform traditional transfer learning approaches for NMSC classification and that a few-shot annotation framework can provide accurate and efficient ROI identification. Future work should focus on further validation in diverse populations, integration into clinical workflows, and addressing the ethical considerations associated with the use of artificial intelligence in healthcare.
